# High uric acid (UA) downregulates bone alkaline phosphatase (BALP) expression through inhibition of its promoter activity

**DOI:** 10.18632/oncotarget.21110

**Published:** 2017-09-20

**Authors:** Zhi-Qi Wu, Xiao-Ting Chen, Yan-Yan Xu, Ming-Jie Tian, Hai-Yan Chen, Guo-Ping Zhou, Hua-Guo Xu

**Affiliations:** ^1^ Department of Laboratory Medicine, The First Affiliated Hospital, Nanjing Medical University, Nanjing, Jiangsu Province 210029, China; ^2^ Department of Pediatrics, The First Affiliated Hospital, Nanjing Medical University, Nanjing, Jiangsu Province 210029, China

**Keywords:** bone alkaline phosphatase, uric acid, promoter, bone metastasis

## Abstract

Bone metastases often occur in prostate cancers, lung cancers and breast cancers. Bone alkaline phosphatase (BALP) is one of the most commonly used serological markers for clinical evaluation of bone metabolism. Here, we reported that high concentrations of uric acid (UA) caused decrease of BALP levels and revealed that the effect of high concentrations of UA on the BALP expression was through inhibition of its promoter activity. Our results suggested physicians to think about serum UA status of patients with advanced cancer to avoid misdiagnosis when BALP was used to diagnose or assess the extent of bone metastases.

## INTRODUCTION

Tumor metastasis is a leading cause of death in patients with advanced cancer [1–3]. Bone is the common site for metastasis in tumor [4, 5]. Bone metastases often occur in prostate cancers, lung cancers and breast cancers [6–12]. Early detection and accurate description of extent of metastatic bone disease is of great significance to the improvement of the quality of life and of the survival time of patients with advanced cancer [13–16]. Bone scintigraphy, the gold standard for monitoring metastatic bone involvement, is highly sensitive in the diagnosis of bone metastases [17–21]. However, it lacks specificity and is not suitable for the patients follow up [22, 23]. In contrast, serological detection has the advantages of early diagnosis, rapid detection and easy continuous monitoring [24–27].

The serological markers of bone metabolism mainly consist of bone formation and resorption markers, such as the bone formation markers tALP, BALP, OC and P1NP and the bone resorption markers BSP, NTX, CTX and TRACP 5b [28–37]. Bone ALP is an 80-kDa glycoprotein, which was found on the surface of osteoblasts. Concentration of BALP generally reflects the rate of bone formation in skeletal tissue [28]. Previously, we reported that the patients with high concentrations of uric acid (UA) presented a false-negative decrease in tartrate-resistant acid phosphatase (TRACP) 5b, a marker of bone resorption, due to a method-related systematic error [38]. It suggested physicians to fully consider interference of hyperuricemia, when TRACP 5b was used for early diagnosis of cancer patients with bone metastasis, evaluation of the aggressiveness of osteosarcoma or prediction of survival in prostate cancer and breast cancer with bone metastases [38]. Considering that BALP is one of the most commonly used serological markers for clinical evaluation of bone metabolism [28–29], we here elucidated the effect of high concentrations of UA exposure on bone metabolism indicator BALP and further revealed its mechanism.

## RESULTS

### Negative correlation between the serum concentrations of UA and BALP

**Figure 1:**
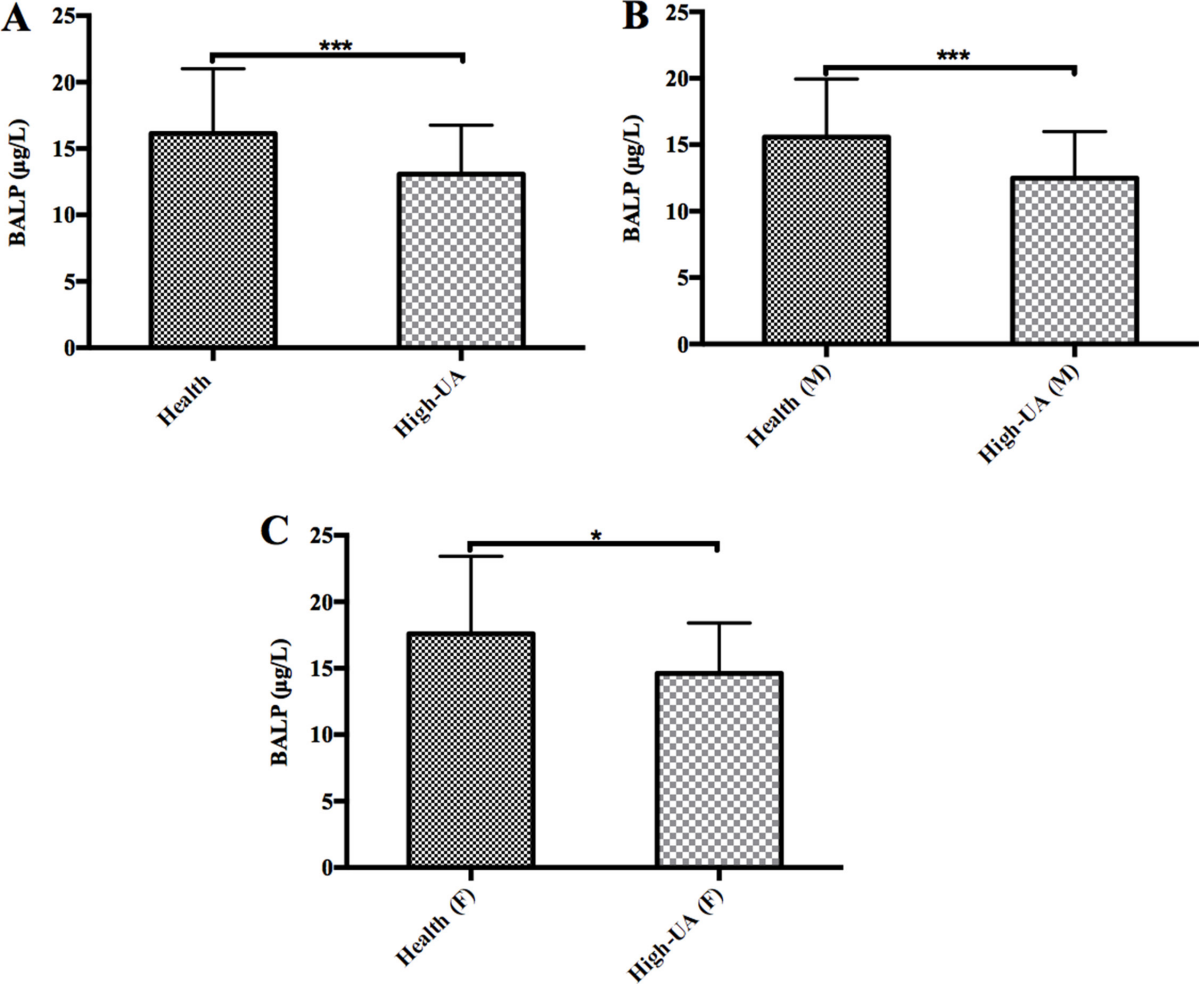
The levels of BALP in the high uric acid groups were significantly lower than the control groups (**A**) The average BALP levels of the 91 high-UA individuals (13.07 ± 3.69 μg/L) were significantly lower (*t*-test, *p* value < 0.0001) than the 91 healthy subjects (16.13 ± 4.87 μg/L). (**B**) The average BALP levels of the 66 male high-UA individuals (12.48 ± 3.50 μg/L) were significantly lower (*t*-test, *p* value < 0.0001) than the 66 male healthy subjects (15.57 ± 4.38 μg/L). Triple asterisk indicates *p* value < 0.0001, and an asterisk indicates *p* value < 0.05. (**C**) The average BALP levels of the 25 female high-UA individuals (14.62 ± 3.78 μg/L) were significantly lower (*t*-test, *p* value < 0.05) than the 25 female healthy subjects (17.59 ± 5.83 μg/L).

Previously, we have reported that high concentrations of UA led to decreased tartrate-resistant acid phosphatase 5b, a marker of bone resorption, in the general population [38]. Here, we checked if BALP, one of the most commonly used serological markers for clinical evaluation of bone metabolism, was affected by high concentrations of UA or not. We randomly tested the BALP levels of 91 high-UA individuals (UA concentration: 485.58 ± 60.39 IU/ml) and 91 healthy subjects (UA concentration: 275.40 ± 64.15 IU/ml). The results showed that the average BALP levels of the high-UA individuals (13.07 ± 3.69 μg/L) were significantly lower (*t*-test, *p* value < 0.0001) than the healthy subjects (16.13 ± 4.87 μg/L) (Table 1) (Figure 1A). The results also indicated that high concentrations of UA led to decreased of BALP levels was not related to sex (Figure 1B, 1C).

**Table 1:** Multiple parameters of serum sample and statistical analyses between groups

Parameter	Study group	Control group	*P* value
**Number of patient (n)**	91	91	/
**Gender (male/female)**	66/25	66/25	/
**Age (years, mean ± SD)**	41.48 ± 16.25	42.78 ± 14.69	0.573
WBC (*10^9^**/L, mean ± SD)**	6.85 ± 1.29	5.95 ± 1.29	0.000***
**NEU % (mean ± SD)**	56.37 ± 8.08	55.28 ± 7.02	0.332
**ALT (U/L, mean ± SD)**	26.41 ± 10.13	20.01 ± 9.21	0.000***
**AST (U/L, mean ± SD)**	22.41 ± 5.29	22.82 ± 5.60	0.611
**AFP (ng/ml, mean ± SD)**	2.93 ± 1.37	3.41 ± 5.79	0.442
**CEA (ng/ml, mean ± SD)**	1.90 ± 1.01	1.84 ± 0.83	0.642
**Glu (mmol/L, mean ± SD)**	5.24 ± 4.67	5.02 ± 4.31	0.001**
**BUN (mmol/L, mean ± SD)**	5.31 ± 1.03	5.08 ± 1.14	0.158
**CREA (μmol/L, mean ± SD)**	76.39 ± 15.69	75.06 ± 14.02	0.548
**UA (μmol/L, mean ± SD)**	485.58 ± 60.39	275.40 ± 64.15	0.000***
**BALP (μg/L, mean ± SD)**	13.07 ± 3.69	16.13 ± 4.87	0.000***

WBC refers to white blood cells, NEU refers to neutrophils, ALT refers to alanine aminotransferase, AST refers to aspartate aminotransferase, AFP refers to alpha fetoprotein, CEA refers to carcinoembryonic antigen, Glu refers to blood glucose, BUN refers to blood urea nitrogen, CREA refers to creatinine, UA refers to uric acid and BALP refers to bone-specific alkaline phosphatase (“**” indicates *p* value < 0.01, “***” indicates *p* value < 0.001).

### Partial removal of UA *in vivo* through renal dialysis led to increase of BALP levels

**Figure 2:**
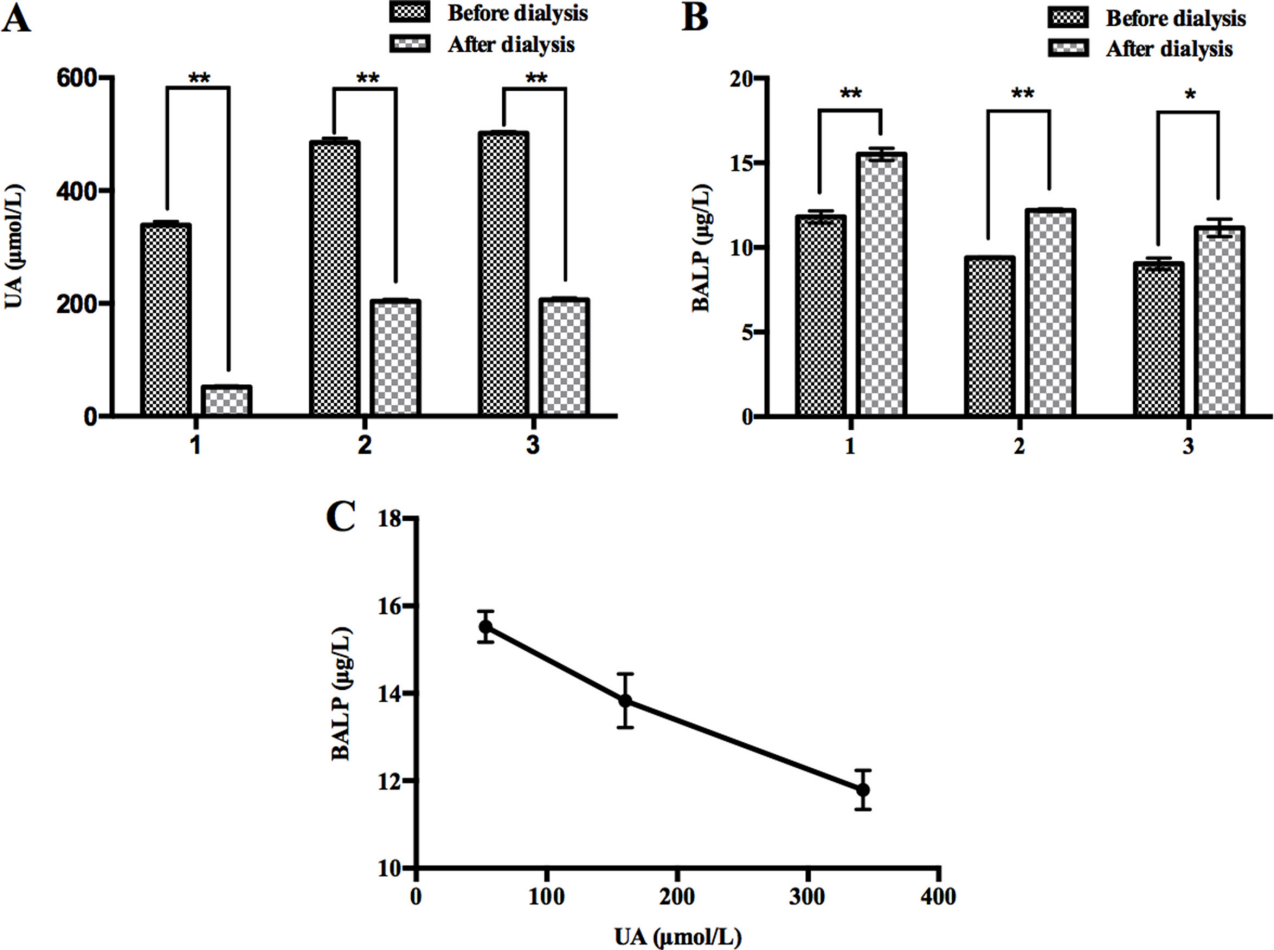
Partial removal of UA *in vivo* through renal dialysis led to increase of BALP (**A**) The levels of UA after hemodialysis were lower than those before hemodialysis in three patients. Double asterisk indicates *p* value < 0.01. (**B**) The levels of BALP after hemodialysis were higher than those before hemodialysis in three patients. Double asterisk indicates *p* value < 0.01, and an asterisk indicates *p* value < 0.05. (**C**) Negative correlation between the concentrations of UA and BALP before, mid and after hemodialysis in No. 1 patient (R = –0.995).

To confirm whether high UA concentrations lead to decrease of BALP levels, we tested the BALP levels of 3 dialysis patients 10 minutes before and 10 minutes after renal dialysis. UA concentrations of these 3 patients after renal dialysis decreased from 342 to 53 μM, 493 to 208 μM, and 498 to 202 μM, respectively (Figure 2A). In contrast, the levels of their BALP increased from 11.8 to 15.5 μg/L, 9.4 to 12.2 μg/L, and 9.0 to 11.2 μg/L, respectively (Figure 2B). In addition, the concentrations of UA and BALP of No. 1 patient among these 3 patients were detected 10 minutes before, half the time of and 10 minutes after renal dialysis. His results showed negative correlation between the concentrations of UA and BALP during hemodialysis (R = –0.995) (Figure 2C). These data suggested that decrease of UA concentrations led to increase of BALP levels.

Based on the above data, we demonstrated that there was a negative correlation between UA concentrations and BALP levels in general population.

### High concentrations of UA did not interfere with BALP immunoassay

**Figure 3:**
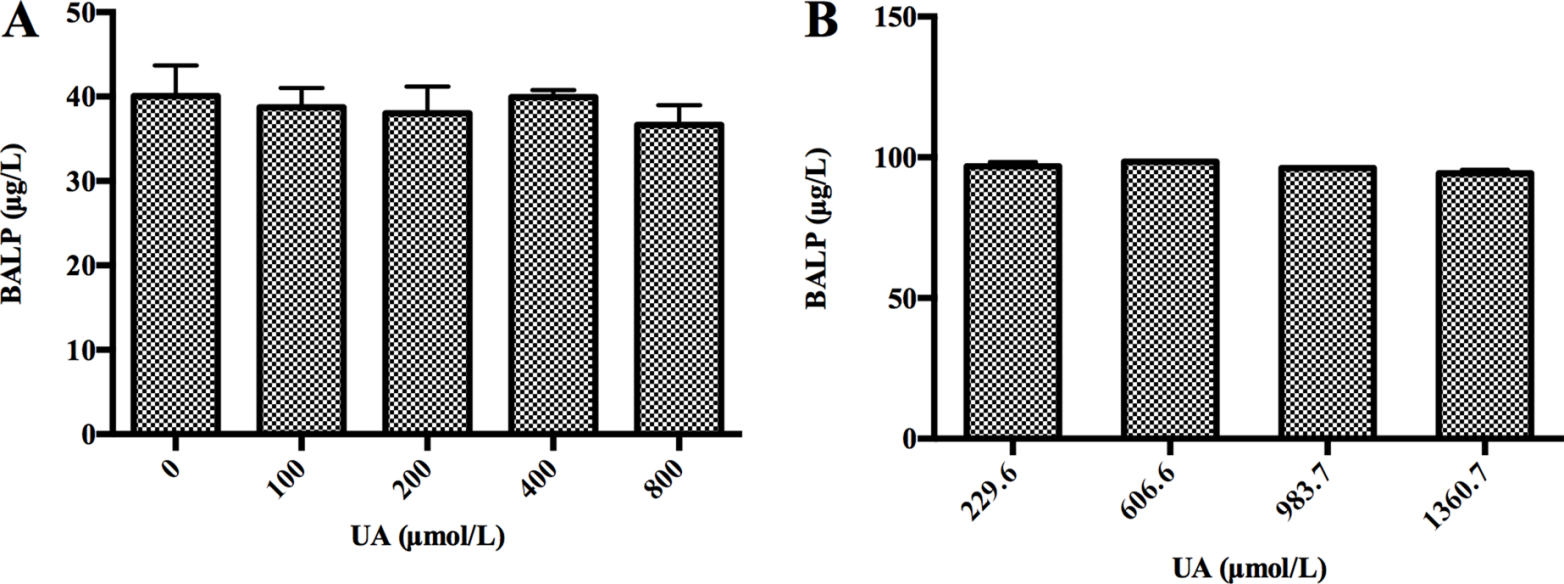
High concentrations of UA did not interfere with BALP level *in vitro* (**A**) High concentrations of UA did not interfere with BALP level of BALP standard samples. A BALP standard sample was divided into 5 aliquots. Serial dilutions of UA (250, 500, 1000 and 2000 µM) were prepared from UA standard subjects. The 4 aliquots were then spiked at 3:2 with each UA solution. This generated 4 different test samples with the same BALP level, whose final UA concentrations ranged from 100 to 800 µM. An aliquot containing DDW instead of UA served as a blank. The average BALP concentration was marked with error bars representing standard deviations of three independent experiments. (**B**) High concentrations of UA did not interfere with BALP level of a serum sample from one patient with confirmed bone metastasis. A UA standard sample was spiked with a serum sample from one patient with confirmed bone metastasis (UA: 229.6 µM, BALP: 96.8 µg/L) at 0:10, 1:9, 2:8 and 3:7, respectively. This generated 4 pools whose final UA concentrations were 229.6, 606.6, 983.7 and 1360.7 µM. The average corrected (multiplied by the dilution) BALP concentrations were marked with error bars representing standard deviations of three independent experiments.

Since high UA concentrations can falsely decrease TRACP 5b levels due to a method-related systematic error, we examined whether high UA concentrations interfered with BALP immunoassay. We performed interference experiments according to the procedure we have described in “Materials and Methods”. We spiked serial dilutions of UA into BALP standard samples and a serum sample from one patient with confirmed bone metastasis, respectively. As a result, we did not observe that BALP levels were affected by gradually increasing doses of UA in BALP standard samples or the patient’s serum sample (Figure 3A, 3B). Therefore, we demonstrated that high UA concentrations did not affect BALP test by interference detection method.

### UA suppressed BALP gene expression through inhibition of its promoter activity

**Figure 4:**
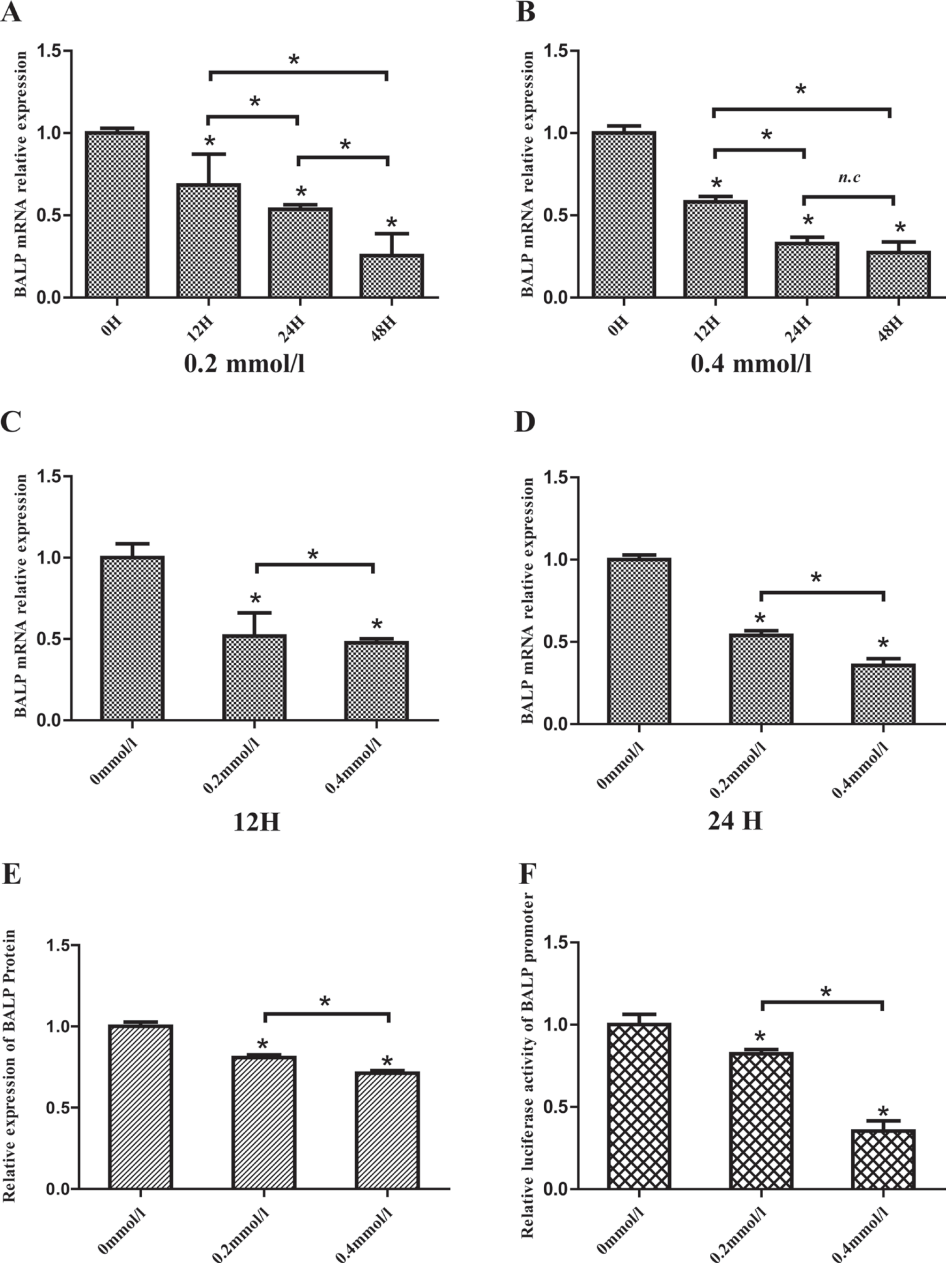
Uric acid suppressed BALP expression in Saos-2 cells (**A, B, C, D**) Human osteoblast-like cells (Saos-2) were vaccinated in 6-well plates and incubated with 0, 0.2 or 0.4 mmol/L Uric acid. Cells were harvested after 12, 24 or 48 hours and the mRNA levels of BALP were measured by qRT-PCR. Data were analyzed using the 2-ΔΔCq method and normalized to GAPDH. Data are represented as mean ± SD of three independent experiments (*p value < 0.05). (**E**) Saos-2 cells were vaccinated in 6-well plates and incubated with different concentrations of UA. The supernatants were collected and the concentrations of BALP were measured. Data are represented as mean ± SD of three independent experiments (*p value < 0.05). (**F**) pGL3-BALP plasmids were transfected into HEK293 cells with pRL-TK using Lipofectamine 2000. After 6 hours, different concentrations (0, 0.2 or 0.4 mmol/L) of UA were added into the mediums. Luciferase activities were measured and the levels of firefly luciferase activities were normalized to the Renilla luciferase activity. Each bar represented the mean ± SD of three independent experiments (*p value < 0.05).

To examine whether high UA concentrations affected the expression of BALP gene, the mRNA levels of BALP were first determined in human osteosarcoma cell line (Saos-2) after treatment with a variety of doses of UA. The results showed that UA treatment decreased the mRNA levels of BALP in dose- and time-dependent manners (Figure 4A–4D). Then, we detected the protein levels of BALP in the supernatant of Saos-2 cells after treated with 200 μM and 400 μM UA for 12h, respectively (Figure 4E).The results indicated that UA treatment also reduced the protein levels of BALP in a dose-dependent manner. To reveal the mechanism, we further checked if high UA concentrations affected BALP promoter activity. We found that BALP promoter activities decreased in HEK293 cells by 27% and 71% after treatment with 200 μM and 400 μM UA, respectively (Figure 4F). These results suggested that high UA concentrations suppressed BALP gene expression through inhibition of its promoter activity.

## DISCUSSION

Uric acid, a metabolic end-product of purine nucleotides, is considered closely related to many kinds of diseases, such as metabolic syndrome, kidney disease, hypertension and cardiovascular disorders [39–43]. As an antioxidant, high UA can decease the production of intracellular ROS and lower the value for glucose as determined by “GOD-Perid” method [44, 45]. Several studies reported that high UA suppresses osteoclastogenesis, affects serum 1,25-(OH)2D3 levels by regulating 1α-hydroxylase activity, and is related to low bone resorption markers [46, 47]. Recently, we reported that high UA concentrations could falsely decrease TRACP 5b, a marker of bone resorption, levels by detection method interference [38]. Here, we checked the effect of high UA exposure on BALP, one of the most commonly used bone metabolism markers, and disclosed its mechanism.

In this study, we first evaluated the differences between a total of 91 patients with high concentrations of UA and 91 healthy subjects. The results showed the average BALP level of the high-UA individuals was significantly lower than the healthy subjects. Meanwhile, we showed that lower UA concentration resulted in increase of the BALP in patients undergoing hemodialysis through renal dialysis. These data suggested that BALP was negatively affected by high UA concentrations, just as TRACP 5b. However, we confirmed that high UA concentrations did not interfere with BALP immunoassay, which was different from TRACP 5b. To disclose how high UA concentrations negatively affected the BALP levels, we detected the mRNA and protein levels of BALP in Saos-2 treated with UA or not. The results showed that high UA concentrations suppressed the mRNA and protein expression of the BALP. Recent researches have shown UA can regulate target genes expression by some different ways. UA induces ET-1 gene expression by the activation of ERK pathway via ROS generation [48]. UA stimulates KHK expression by binding to a specific sequence within its promoter [49]. High UA increases KLF2 expression by miR-92a downregulation [50]. Here, we confirmed that the effect of high UA on the BALP expression was through inhibition of its promoter activity.

As we know, BALP was widely used serological marker in the detection of bone metabolism. Many studies used BALP to assess bone growth and the extent of bone metastasis [51–56]. However, we did not find that UA status of the patients in these studies was considered. Tumor could promote hyperuricemia through tumor related cell death, due to tumor treatments [57]. Therefore, the reliability of previous research results based on BALP without considering serum UA status of patients needs further confirmation. Our results suggested physicians to think about serum UA status of patients with advanced cancer including lung cancer, breast cancer and prostate cancer to avoid misdiagnosis when BALP was used to diagnose or assess the extent of bone metastases.

Although we have made clear that high UA concentrations negatively affected TRACP and BALP levels, two widely used bone metabolic markers, we do not know whether other bone metabolism markers such as OC, NTX and CTX are affected by high UA concentrations. We will further disclose whether serum UA status have effects on the level of other bone metabolic markers in our future work to decrease the misdiagnosis rate of bone metabolism.

## MATERIALS AND METHODS

### Serum sampling

This study was approved by the Ethics Committee of the First Affiliated Hospital of Nanjing Medical University. All samples were collected from August 2014 to February 2016. Patients with cancer, hepatitis, renal dysfunction and inflammatory disease were excluded from serum collection. A total of 91 patients, including 25 women and 66 men (median age, 41 yr; range, 22–88 yrs) formed the study group. Additionally, 91 healthy subjects were sampled as the control group, including 25 women and 66 men (median age, 42 yr; range, 22–89 yrs).

### Data collection

The ALT, AST, Glu, UREA, CREA and UA quantitation were analyzed using an Olympus AU5400 automatic chemical analyzer and commercial kits (Olympus, Japan) according to the instruction manual. WBC and NEU % were counted by the Sysmex XE-2100 hematology analyzer (Sysmex, Kobe, Japan). The levels of CEA and AFP were measured by electrochemiluminescence immunoassay (ECLIA) on an Elecsys E 602 (Roche Diagnostics, Basel, Switzerland). Serum BALP was detected by using Unicel DxI 800 (Beckman Coulter, USA) device via the chemiluminescence enzyme immunometric method.

### UA interference experiment

(1) A known concentration of BALP standard sample was divided into 5 aliquots. Serial dilutions of UA (250, 500, 1000 and 2000 μM) were prepared from UA standard subject. The 4 aliquots were then spiked at 3:2 with each UA solution. This generated 4 distinct test samples with the same BALP concentration, final UA concentrations ranging from 100 to 800 μM. An aliquot containing double distilled water (DDW) instead of UA served as a blank. Then measured the BALP concentrations of each aliquot. (2) A UA standard sample was spiked with a serum sample from one patient with confirmed bone metastasis (UA: 229.6 µM, BALP: 96.8 µg/L) at 0:10, 1:9, 2:8 and 3:7, respectively. This generated 4 pools whose final UA concentrations were 229.6, 606.6, 983.7 and 1360.7 µM. Then measured and corrected (multiplied by the dilution) BALP concentrations of each aliquot.

### Cell culture

The human osteoblast-like cells (Saos-2) and human embryonic kidney 293 cells (HEK293) were obtained from ATCC and maintained in DMEM (Gibco, USA) supplemented with 10% fetal bovine serum (Gibco, USA) with 1% penicillin streptomycin, at 37°C in 5% CO_2_, harvested by trypsinization, and subcultured twice weekly.

### Quantitative real-time PCR (qRT-PCR)

Saos-2 cells were seeded at a density of 200,000 cells/well in 6-well dishes. Cells were grown in DMEM with 10% FBS, and then 24 hour later incubated with 0, 0.2, 0.4 mmol/L UA (*Sigma*-Aldrich, USA) for 0, 12, 24 or 48 h. Total RNA of cells was extracted with Trizol reagent (Life technologies, UK) according to manufacturer’s instructions. cDNA was transcribed from total RNA using PrimeScript™ RT Master Mix (Perfect Real Time) (TAKARA, JAPAN). The mRNA level of BALP and GAPDH were quantified by real time-PCR using SYBR^®^ Premix EX Taq™ (Tli RNaseH Plus) (TAKARA, JAPAN) in ABI 7300 real-time PCR system (Applied Biosystems Inc.). The forward and reverse primer sequences were designed using Olige 7 software and listed in Table 2. The GAPDH gene was used as an internal standard. The specificity of amplification was assessed by melting curve analysis for each sample. The ΔΔCt method was used to transform Ct values into relative quantities (mean ± standard deviation). Changes were expressed as a percentage of the controls.

**Table 2:** Sequences of oligonucleotides used to clone BALP gene promoter and qRT-PCR amplifications

Names	Primer sequence (5′–3′)	
	Forward	Reverse
**pGL3-BALP**	5′ GG*GGTACC*GTGCAGAGTCAGAGGTGCACGT 3′	5′ GA*AGATCT*GAGCACTGGCGAGGGTCCGTCC 3′
**GAPDH**	TGCACCACCAACTGCTTAGC	GGCATGGACTGTGGTCATGAG
**BALP**	GGACCCTCGCCAGTGCT	GTGCACCCCAAGACCTGC

Primers named pGL3-BALP for cloning promoter of BALP gene: *Kpn* Ⅰ restriction site (underlined and italics) and protective bases were introduced to the 5′ end of the forward primers, and *Bgl* Ⅱ restriction site (underlined and italics) and protective bases were added to the 5′ end of the reverse primers. Primers named as GAPDH and BALP for qRT-PCR.

### Generation of report plasmid constructs

A 5′ flanking upstream (including promoter region) of BALP (NM_000478.5) (from -473 to +154 nt relative to transcription start site) was amplified from genomic DNA by PrimeSTAR^®^ HS DNA Polymerase (TAKARA, JAPAN) according to manufacturer’s protocol. The forward and reverse primers were designed using Olige 7 software and listed in Table 2. The PCR products were digested with *Kpn I* and *Bgl II* (Thermo Fisher Scientific, USA) and then subcloned to a promoter-less vector pGL3-Basic restrictively digested with *Kpn I* and *Bgl II*. The recombinant plasmids were tested and sequenced, and the positive cloned plasmid was named as pGL3-BALP.

### Transient transfections and luciferase assays

Transient transfections were carried out in HEK293 cells using Lipofectamine2000 (Invitrogen, USA) according to the manufacturer’s suggestion. Cells were vaccinated in a 48-well culture plates and when the cells had grown to 70–80% before transfection. For luciferase assays, 200 ng of pGL3-BALP plasmid was cotransfected into HEK293 cells with 2 ng of control pRL-TK plasmids as an internal control. After 6 hours, cells were incubated with different doses (0, 0.2, 0.4 mmol/L) of UA, respectively. After 24 hours, cells were harvested and luciferase assays were measured with the Dual Reporter assay system (Promega, USA) using FB12 luminometer (Berthold, Germany). The relative luciferase activities (RLA) were calculated by normalizing the fluorescence luciferase with internal standard Renilla luciferase.

### Statistical analysis

The statistical software package SPSS17.0 (SPSS Inc, Chicago, USA) was used for data analysis. Data were presented as means ± SD and represented at least three independent experiments. Two-tailed *t*-tests were used for significance testing between groups of continuous data. For all statistical comparisons, a *p* value < 0.05 was considered statistically significant.

### Ethical standards and patient consent

Ethical clearance for this study was obtained from the Ethics Committee at the First Affiliated Hospital of Nanjing Medical University. Because all the samples used in this study were collected from clinical residual specimen, written informed content from each patient was waived. This study was conducted in accordance with the Declaration Helsinki.
